# Development of biodegradable film from cactus (*Opuntia Ficus Indica)* mucilage loaded with acid-leached kaolin as filler

**DOI:** 10.1016/j.heliyon.2024.e31267

**Published:** 2024-05-21

**Authors:** Alebel Abebaw Teshager, Minaleshewa Atlabachew, Adugna Nigatu Alene

**Affiliations:** a Faculty of Chemical and Food Engineering, Bahir Dar Institute of Technology, Bahir Dar University, P.O. Box 26, Bahir Dar, Ethiopia; b Department of Chemistry, College of Science, Bahir Dar University, P.O. Box 79, Bahir Dar, Ethiopia

**Keywords:** Cactus peer (*Opuntia Ficus Indica*), Mucilage, Bioplastics, Acid leached kaolin, Plasticizer

## Abstract

Nowadays, substituting petroleum-based plastics with biodegradable polymers made from polysaccharides loaded with various reinforcing materials has recently gained attention due to the impact of conventional plastics wastes. In this study, polysaccharidic mucilage from Ethiopian cactus (Opuntia Ficus Indica) was derived using microwave-assisted extraction technique to develop biodegradable polymers that were inexpensive, readily available, simple to make, and ecofriendly. The effect of microwave power 300–800 W, solid-liquid (cactus-sodium hydroxide solution) ratio 1:5–1:25, sodium hydroxide concentration 0.1–0.8 mol/L, and extraction time 2–10 min on mucilage extraction were studied and the maximum yield of mucilage was attained at optimized parameters of 506 W, 1:20, 0.606 mol/L, and 9.5 min, respectively. Biodegradable polymers made with mucilage alone have poor mechanical characteristics and are thermally unstable. Thus, to overcome the stated problems, glycerol as a plasticizer and acid-leached kaolin crosslinked with urea as a reinforcing material were used. Moreover, the effect of acid-leached kaolin and glycerol on the physico-chemical properties of the films was studied, and a maximum tensile strength of 6.74 MPa with 18.45 % elongation at break, thermally improved biodegradability of 26 %, were attained at 10 % acid-leached kaolin and 20 % glycerol crosslinking with 2 % urea. But the maximum degradability of 53.5 % was attained at 30 % glycerol content. The control and reinforced biodegradable films were characterized using TGA, FTIR, SEM, and XRD to determine the thermal, functional group, morphology, and crystallinity of the bioplastics, respectively. These biodegradable plastics may be used for packaging application.

## Highlights

• Mucilage based Biodegradable films reinforced with acid leached-kaolin have been prepared.
• Mucilage has been extracted by using micro wave assisted extraction technique.
• Reinforcing material improves the mechanical and thermal properties of the film.
• The process variables on mucilage extraction was optimized.

## Graphical abstract

**Figure:**
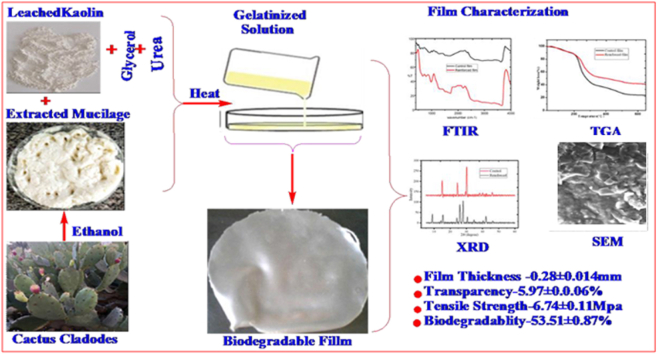

## 1 Introduction

The global plastic production is growing exponentially reaching 380 million tons per year in 2018 [1], with a forecast of 33 billion tons by 2050 [2,3]. High mechanical strength, chemical durability and low production costs make plastic products ubiquitous in all areas of human life [4]. As such, plastic products are used in every aspect of daily life, such as food and product packaging, textiles, building materials and automobiles, home appliances, medical equipment, agriculture, personal care products, toys, engineering as well as research fields [1,2,5]. Almost 40 % of global plastic production is used for plastic packaging (67 % by weight in 2020) in perishable goods, including food and beverages [[6], [7], [8]]. Due to the huge production of these plastics, around 100 million tons of plastics are generated/dumped as waste in to the environment and poses socio-economic and environmental hazards including all living organisms (aquatic life), reducing landfills, and lowering soil fertility [9]. Hence, in the past, researchers have proposed incineration and recycling of plastic wastes as promising solutions to minimize such impacts. However, these options may lead to other problems for a sustainable environment by releasing toxic gases and damaging the world's economy and labor resources [10]. Incineration also contributes to the problems of global warming and acid rain [9].

Recently, the demand for degradable plastics, especially biodegradable plastics, has been growing due to the influence of petroleum based plastic wastes [11]. The degradation can happen through either physicochemical and/or biological processes: [10]. Physicochemical processes include weathering (degradation due to sunlight, wind, or waves) and hydrolysis/oxidation [10], but such degradation may lead micro/nanoplastics (MNPs) formation and cause health impacts on living organisms [12]. While biodegradation can occur via microbial agents, they use carbon as food and generate biomass, carbon dioxide, and water as by-products [10] and during thus, the microorganisms first cleave the polymer chains to penetrate easily into the cells and then degrade the polymeric materials by excreting extracellular enzymes that depolymerize the polymers [13].

As such, the synthesis of biodegradable plastics from agricultural wastes/biomasses has gained attention year by year due to their cost effectiveness, availability, biodegradability and environmentally benign properties [14,15]. Biodegradable films can be developed from various polysaccharides that are found in various agricultural wastes such as starch from mango seed kernel [14], cellulose from sugarcane bagasse [16], protein-based [17], mucilage from cactus [18] and more. However, polysaccharide based biodegradable films are exposed to low thermal and mechanical properties [19] due to the presence of numerous hydroxyl groups and makes the film more hydrophilic, which affects the practical application of biodegradable films [14]. To overcome such shortcomings, incorporating organic and inorganic reinforcing materials into the polymer matrix has been adopted by the scientific community [20,21]. Thus, the interaction of polysaccharide with reinforcing materials can produce strong inter- and intramolecular interactions via hydrogen bonding, which enhances the mechanical as well as thermal properties and lowers the water affinity ability of biodegradable films [14,22].

**Fig. 1:**
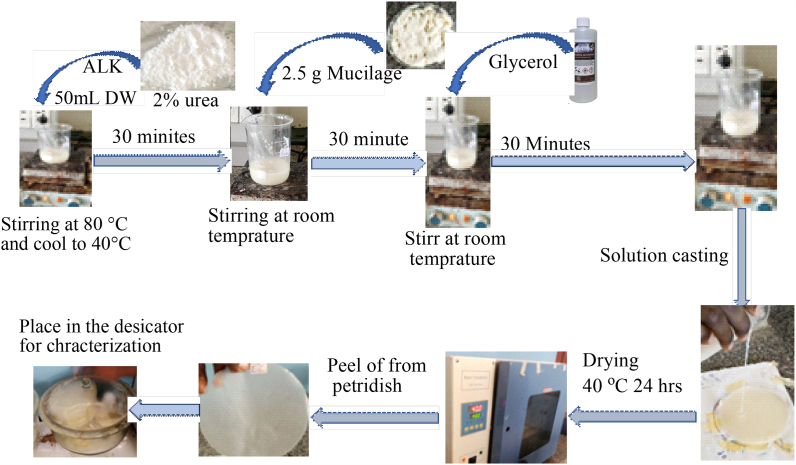
Flow diagram for preparation of a biodegradable film.

This study was used Ethiopian cactus (*Opuntia Ficus Indica*) cladode (Fig. 1) as a source of mucilage-polysaccharide to develop biodegradable packaging films. Cactus (*Opuntia Ficus Indica*) is a family Cactaceae and its fruit used as a food source due to its high nutritional value [23] and cladodes as agrowaste used for animal feed in some areas [23]. The cladodes are rich in mucilage (a complex polysaccharide that contains several sugar units, and it has stabilizing, emulsion and foaming as well as gel formation ability) [24] and used for developing biodegradable film for packaging applications [18]. Ethiopian cactus is mostly found in most areas of the country, especially in Tigray (covers more than 379,338 ha of land), Amhara and Oromia regions [25]. As such, in addition to their highly availability/affordability and cost effectiveness of the cactus fruits, the cladodes are disposed or dumped into the environment as an agro waste. Moreover, Ethiopian cactus cladode mucilage were not investigated regarding alternative solution for manufacturing industries as well as packaging applications. Hence, this finding used a cladodes agro waste as a potential source of mucilage by (microwave-assisted extraction) to develop biodegradable packaging materials. However, biodegradable plastics made from mucilage are fragile in their mechanical properties and thermally unstable. To overcome such limitations, we have used acid-leached kaolin, which is cost-effective, affordable (readily available), and environmentally friendly, as well as urea as an environmentally favorable crosslinking agent, to develop enhanced mucilage-based biodegradable film. Furthermore, the films were characterized using Fourier transform infrared spectroscopy (FTIR), thermogravimetric analyzer (TGA), scanning electron microscopy (SEM), and x-ray powdered diffractometer (XRD).

## 2 Materials and methods

### 2.1 Materials

All chemicals/reagents were of analytical grade and utilized without further purification. Distilled water was used to prepare all the analyte solutions. Ethanol (99 %), potassium sulphate (99 %) and oxalic acid (99 %); sodium hydroxide (98 %), hydrogen peroxide (10 %) and sulfuric acid (98 %); hydrochloric acid (37 %), urea (98 %), glycerol (99 %) and copper sulphate (99 %) were obtained from India MART, Lobachemie and Sigma Aldrich, respectively. Methyl red, petroleum ether and local kaolin were also used.

Instrumentation: Drying oven/incubator (PH-030A), China), muffle furnace (Nabertherm GmbH, Germany), UV–visible spectrophotometer (lambda 35, PerkinElmer, Germany), tensile strength tester (TENSOLAB 1000,MESDAN, Italy), digital micro meter (7301, Mitutoyo, Japan), blender (ZAIBA, ZA-728*,* China), microwave oven (SYinix, MW1023-02D), Fourier transform infrared spectrometer, FTIR (FT/IR660, JASCO, Japan), thermogravimetric analyzer, TGA (HCT-1, BJHENEVEN, China), Scanning Electron Microscope, SEM (JCM-6000Plus), and X-ray Diffractometer, XRD (XRD-7000, SHIMADZU, Japan) were used in this analysis.

### 2.2 Sample collection and preparation

The cactus cladodes were collected from Simada Woreda (around 200 km from Bahir Dar) and kaolin was taken in the research-grade laboratory of the Faculty of Chemical and Food Engineering, Bahir Dar Institute of Technology, Bahir Dar University, Ethiopia. Then, the spine of the cladode was carefully removed by knife without losing the internal part and made ready for peeling by washing with distilled water several times to remove impurities. While kaolin was crushed with a miller and allowed in between 63 and 250 μm sizes and ready for leaching.

### 2.3 Microwave assisted extraction of mucilage

Extraction of polysaccharidic mucilage from cactus cladodes were presented according to Ref. [26] with slight modifications. The spinless cladode was washed with distilled water, peeled, and was taken the inner part. Briefly, 10 g of the inner part (peeled) of cladode was homogenized using a blender for l minutes to get a uniform cladode size. Then, the homogenized cladode was mixed with sodium hydroxide solution by varying the concentration (0.1 M, 0.45 M, and 0.8 M) with solid to liquid ratio 1:5, 1:10, and 1:25 by following the design expert software response surface methodology (RSM) of the central composite design (CCD) run order. After this, the conical flask that contained homogenized cladode mixed with sodium hydroxide solution was placed in to microwave oven by varying the power to 300, 500 and 800 W for the required time 2, 6, and 10 min based on experimental design run order. Also, similar procedure was applied for 30 experiments with duplicate and the independent variables microwave power, concentration, solid-liquid ratio and extraction time were optimized by considering mucilage yield as a response. After microwave extraction was completed, the mixture was filtered using nylon cloth and precipitated using ethanol (95 %) with mucilage extract to ethanol ratio of 1:3 v/v (Fig. 3). The precipitate was filtered using a vacuum filter and washed with hydrogen peroxide (10 %) to bleach and ethanol (75 %) in distilled water several times. Finally, the precipitated mucilage was dried (50 °C), calculated the yield and stored for further analysis.

### 2.4 Physicochemical characterization of mucilage

For pH determination, the mucilage was diluted with distilled water (1:100w/v) [27] and the supernatant was directly measured by a pH meter (PHS–3B, China). Water-holding capacity (WHC) of mucilage was determined by using the method [28] with some modifications. Briefly, 0.2 g mucilage powder sample was mixed in 10 mL distilled water with stirring for 1 min and centrifuged at 3000 rpm for 30 min. Then, the supernatant was removed and the final mass was weighed and water-holding capacity of mucilage was calculated. Solubility and swelling power were determined according to Ref. [29] with slight changes. A sample of 0.25 mucilage was immersed in 10 mL distilled water in a beaker, heated on hot plate at 65 °C for 10 min and it was in cold water for 5 min. Next the mixture was centrifuge at 3000 rpm for 15 min and the supernatant was dried in an oven at 105 °C until constant weight reaches. The precipitated paste and the dried supernatant were weighed and the solubility and swelling power were calculated. Moisture content of powder mucilage (2 g) was determined by drying in an oven at 105 °C for 2 h [27] and ash content was determined by taking 2 g of mucilage powder at 600 °C for 8hrs [30]. Protein content was estimated using the Kjeldahl method (N × 6.25) [27]. Fat (or lipid) content was determined using Soxhlet extraction method using petroleum ether as a solvent [31]. After calculating moisture, protein, fat, and ash the amount of carbohydrates was calculated by subtracting from the total dry matter [30,32].

### 2.5 Preparation of kaolin

The local kaolin powder was soaked with distilled water (1:10) for 24 h in a 1000 mL beaker, and the upper layer was decanted to get remaining kaolin. Next, the remained kaolin was dried in a drying oven at 105 °C for 6 h. The dried kaolin was treated with 2 M oxalic acid (1:10) to remove impurities (feldspar, quartz, mica, muscovite, and iron oxide), placed and stirred at 300 rpm and 80 °C on a hotplate for 3 h. Finally, acid leached kaolin was washed with distilled water until the pH became neutral, and dried in an oven at 105 °C for 24 h [33].

### 2.6 Preparation of biodegradable films

The mucilage based biodegradable films were developed according to the previous reports [34,35] with slight modifications. The required amount of acid-leached kaolin (0 %, 5 %, 10 %, 15 %) and urea (2 %) as a crosslinking agent were mixed with 50 mL of distilled water following a full factorial experimental design. It was stirred at 80 °C for 30 min and then cooled to 40 °C. Then, 2.5 g of powder mucilage was added into the cooled solution and stirred for 30 min at room temperature followed by glycerol as plasticizer (0 %, 10 %, 20 %, 30 %) and stirred for another 30 min to homogenize the mixture at room temperature. Finally, the solution was casted on a plastic Petri dish and dried in a ventilated oven at 40 °C for 24 h and peeled from the Petri dish and kept in a desiccator till for further analysis and the over all process is illustrated as shown in Fig. 1.

### 2.7 Characterization of the mucilage based Biodegradab1e film

#### 2.7.1 Moisture content and water solubility

The moisture content and water solubility of the films were determined using the previous method [14]. For moisture content, film samples (2 cm × 2 cm) were oven-dried at 90 °C for 24 h. The solubility of the film was determined by taking film samples (2 cmx2 cm), which were dried in the oven at 80 °C for 24 h, cooled to room temperature in a desiccator, weighed (initial mass), and then immersed in 50 mL of distilled water at 25 °C for 6 h. The undissolved pieces were oven dried at 90 °C for 24 h and then weighed (final mass) after cooling to room temperature and calculated the film's water solubility.

#### 2.7.2 Thickness and transparency of films

The thickness and transparency of the films were determined according to the literature [36]. The thickness of the film was determined by using a digital micrometer at four locations on the film, and the average value was reported. While film transparency was determined spectrophotometrically, film samples were cut to a size of 1 × 4 cm and put into a quartz cuvette and an empty quartz cuvette served as a blank and conducted the measurement at a wavelength of 620 nm.

#### 2.7.3 Biodegradability

The biodegradable behavior of the developed films based on full factorial design order was determined using the ASTM D5988 soil burial technique [14]. The film was cut into 5 × 5mm^2^ and then buried in the ground at 100 mm depth for 30 days. Before burial, the initial weight was determined. At the end of the testing period (30 days), the film materials were removed from the soil, washed with distilled water to eliminate adherent soil, and dried on filter paper. The samples were stored in the desiccator for 24 h until they reached a constant weight. The final mass of samples was weighed and the degree of degradability (%W) was calculated.

#### 2.7.4 Tensile strength and elongation at break

The tensile strength and elongation at break of films were determined by using universal testing machine according to the ASTM D882-10 [14] at a crosshead speed and gauge length of 50 mm/min and 50 mm, respectively. The width of all samples was taken as 1 cm and the equipment was generated both maximum force and elongation at break simultaneously.

#### 2.7.5 Surface functional groups (FTIR) analysis

The surface functional groups of mucilage and mucilage-based films were interpreted using FTIR spectroscopy in the range of 400-4000 cm^−1^ with a resolution of 4 cm^−1^. Briefly, 1 mg of mucilage powder were mixed with potassium bromide (1:100), homogenized using mortar and pestle, pelletized by mechanical press. For mucilage-based films, the films were cut in to 4 cm and scanned directly using FTIR.

#### 2.7.6 Thermal (TGA) analysis

The thermal properties of mucilage-based films were determined by using a thermogravimetric analyzer (TGA) in the range of room temperature to 700 °C with a heating rate of 20 °C/min and a nitrogen air flow rate of 20 mL/min. The thermal properties of mucilage were also characterized in the range of room temperature to 900 °C under similar conditions.

#### 2.7.7 Determination of crystallinity and morphology of films

The morphology of biodegradable films was determined using a scanning electron microscope (SEM). A small piece of the biodegradable film was cut using scissors, and the specimen stub with the coated sample is placed into the SEM chamber. The SEM images of the film were captured at ×1500 magnification and with a beam voltage of 10 kV for both films. While the crystallinity of the films was analyzed using an X-ray diffractometer (XRD) in the scanning range of two theta 5–60 at a voltage of 40 kV and 30 A with a scan speed of 3.00°/min, The peak area in the crystallinity and for both amorphous and crystallinity of the films were calculated using Origin Pro software 2019 and crystallinity index for the films were estimated [37].

## 3 Result and discussion

### 3.1 Stastical analysis on mucilage extraction

Design Expert (Stat-Ease, Inc., version 13.0.0, Minneapolis, USA) software with Response Surface Methodology (RSM) through three levels with face-centered by central composite design (CCD) was used for statistical data analysis and experimental design to see the effect of microwave power, concentration of sodium hydroxide, solid-liquid ratio, and extraction time on mucilage yield. The effect of independent process parameters such as microwave power (A), solid-liquid ratio (B), concentration of sodium hydroxide (C), extraction time (D) and mucilage yield as a response was investigated by using this software. About 30 experiments were conducted based on design run order. The statistical analysis revealed that the experimental values fit the second-degree polynomial equation and the model suggested by this central composite design (CCD) was quadratic based on maximizing adjusted R^2^ and predicted R^2^ values (Table 1).

**Table 1:** Model summary of statical analysis.

Source	Sequential p-value	Lack of Fit p-value	Adjusted R^2^	Predicted R^2^	
**Linear**	0.0075	<0.0001	0.3222	0.1624	
**2FI**	0.9886	<0.0001	0.1464	1.1316	
Quadratic	**< 0.0001**	**0.0616**	**0.9925**	**0.9793**	**Suggested**
**Cubic**	0.1183	0.1075	0.9959	0.8736	Aliased

Additionally, the central composite design (CCD) ANOVA analysis for quadratic model analysis was used to determine the significance of independent variables and their interactions with yield. The model F-value of 343.91 implies the model is significant. There is only a 0.01 % chance that an F-value this large could occur due to noise. *P*-values less than 0.0500 indicate that model terms are significant. In this case, A, B, C, D, AD, BC, BD, A^2^, B^2^, C^2^, D^2^ are significant model terms. *P*-values greater than 0.05 were reduced or not considered significant factors.

**Fig. 2:**
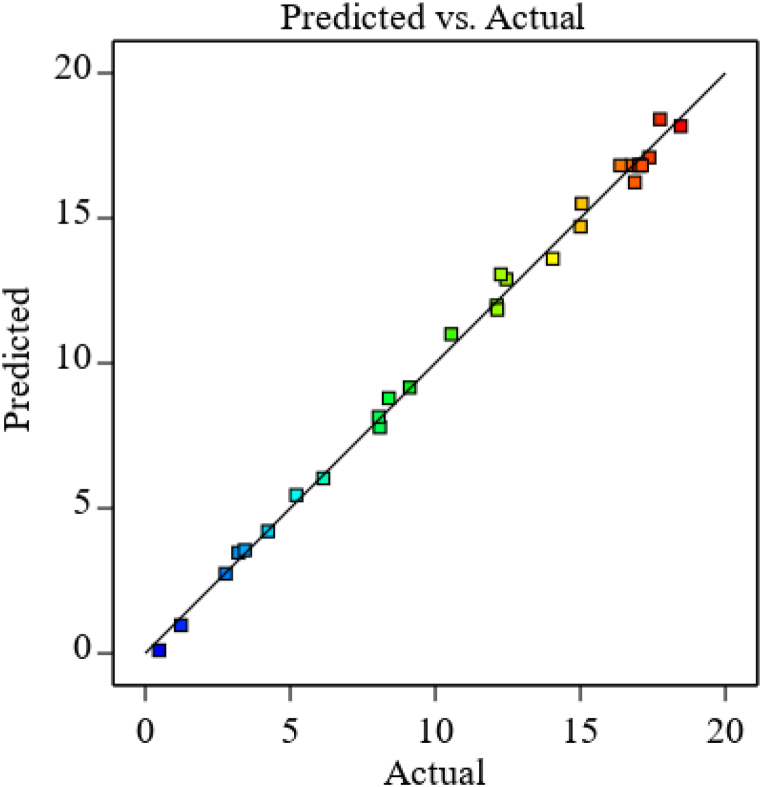
Actual vs predicted values on extraction of mucilage.

The actual (experimental value) and predicted values were also almost perfectly fitted with the fitting line. This may validate that the experimental results are generally distributed close to a straight line, indicating strong model-experiment data fit (Fig. 2).

**Fig. 3:**
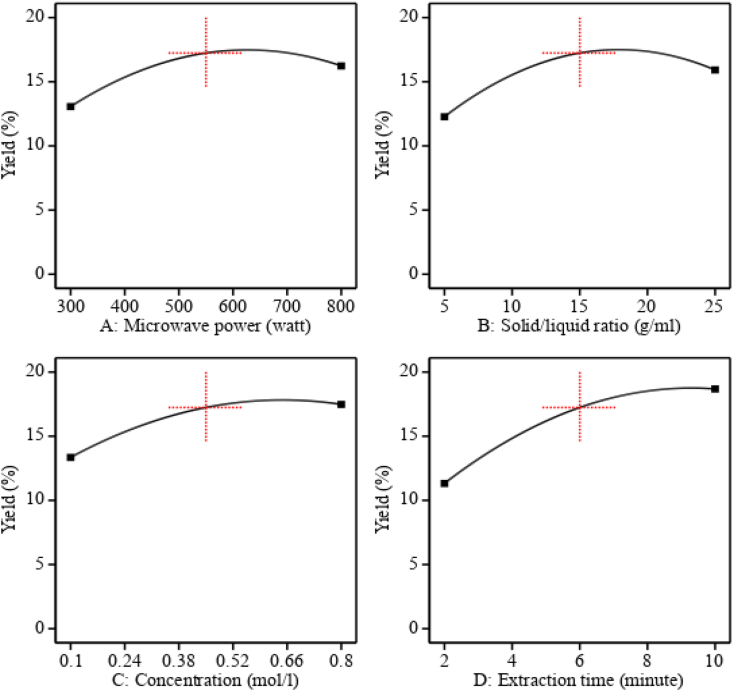
Effect of process parameters on mucilage extraction yield.

Additionally, the software provides a coded equation to predict how independent process factors would impact dependent variables, as shown below.

$$\text{Yield}=17.24+1.58A+1.82B+2.07C+3.6D+0.4851AD+0.4031BC+1.02BD-2.59A^{2}-3.1B^{2}-1.82C^{2}-2.23D^{2}$$

The equation in terms of coded factors can be used to make predictions about the response at given levels of each factor. The coded equation is useful for identifying the relative impact of the factors by comparing the factor coefficients. Based on this, the main factor was extraction time, which had a factor coefficient of 3.6, followed by the square of microwave power and the concentration of sodium hydroxide. Since the reduced yield obtained with lower values of other parameters can be improved by increasing the extraction time, the degradation of the desired component that can occur with maximum values of other parameters may be mitigated by reducing the extraction time. Therefore, controlling the extraction time is a better responsibility for improved mucilage extraction. From this coded equation, the linear equation (A, B, C, and D) and the interaction effect (AD, BC, and BD) affected the mucilage yield positively. The analysis of variance also indicated that the interaction effect of (AD, BC, and BD) affect significantly the mucilage yield. When all parameters are at their maximum value, the quadratic equation has a negative effect, which implies the desirable component may be exposed to deterioration, which lowers yield. Therefore, the square of all these parameters may affect the mucilage yield negatively.

### 3.2 Factors affecting cactus mucilage extraction

In this study, the effect of microwave power (300–800), solid-liquid ratio (1:5–1:25), concentration of sodium hydroxide (0.1–0.8 M), and extraction time from 2 to 10 min on mucilage yield were investigated by reviewing numerous literatures and conducting preliminary work to decide the working range of those parameters. Based on the analysis of variance response, the microwave power significantly (p < 0.05) affects the mucilage extraction yield, and the result showed that when the microwave power increased from 300 to 600 W, the yield of mucilage was enhanced as shown in Fig. 3(A). Because the increased microwave power makes it easier for the solvent to penetrate the plant matrix by accelerating molecular movement, which increases the yield of mucilage. Furthermore, when the power of the microwave increased, the surface area between the plant matrix and the solvent also increased due to the disruption of the plant's cell walls, which increased the yield [26]. Even though, the mucilage yield decreases as the microwave power are raised above 600 W, this is because of the microwave radiation's maximum intensity, which may cause the desirable components in the plant matrix to degrade. The maximum amount of yield was attained at 506 W microwave power and the result was in agreement with the result of [26] with similar plant species.

Moreover, the effect of solid-liquid ratio on mucilage extraction was investigated from 1:5 to 1:25 following the design expert run order. The analysis of variance (p < 0.05) result showed that the solid-liquid ratio had a significant impact on the yield of mucilage, and the results confirmed that as this ratio increased, the extraction of mucilage also improved because the solvent's diffusivity and ability to penetrate the cladode cell matrix increased, which led to an increase in the elimination of the desired component from the cladode matrix [38]. Furthermore, promoting a solid-to-liquid ratio might reduce mucilage's stickiness due to a greater amount of solvent, which may improve the effectiveness of mucilage extraction. Though increasing the maximum amount of solvent may have a cost impact, the yield started to reduce after the 1:20 ratio of cladode to sodium hydroxide solution, as shown in Fig. 3(B). Since the maximum solid-liquid ratio may lead to a higher concentration difference between the inner cladode cells and the extraction solvent, which may increase the diffusion of polysaccharides and, as a result, reduce the yield. Because at greater solid-to-liquid ratios, the desired component is eliminated during the filtration process along with undesirable liquids. Therefore, the appropriate solid-to-liquid ratio is needed for improved extraction yield, and in the present study, the maximum yield was noted at a solid-to-liquid ratio of 1:20, keeping other variables constant. This result strongly deviated from the result of [39] on polysaccharide extraction with similar extraction techniques, a range of solid-liquid ratios, and different species, this may be due to the variety of plants and environmental factors.

The concentration of sodium hydroxide has a significant role (p < 0.05) in the extraction of polysaccharides from various plant matrixes [40]. In this study, the effect of sodium hydroxide concentration (0.1–0.8 mol/L) on the mucilage yield was investigated. The results indicate that as the concentration of alkaline solvent improves, the yield of mucilage also increases. This is because the cladode cell walls' intermolecular interactions are broken, which enhances the removal of the mucilage from the cladode matrix. As seen in Fig. 3(C)–as the concentration increases from 0.1 to 0.8 mol/L, the yield also improves. However, beyond 0.6 mol/l, the yield begins to decrease, which may be caused by the desired component, mucilage, degrading at higher concentrations of alkaline solvent or causing polysaccharide hydrolysis. The lowering of concentration did not extract mucilage, and increasing concentration changed the fundamental structural composition of mucilage. Therefore, the optimum value of concentration plays a crucial role in the extraction of mucilage from cladodes. Hence, 0.66 M of NaOH was found to be optimal for this experiment. This result is in agreement with the previous study [40].

Furthermore, the effect (p < 0.05) of irradiation time or extraction time on mucilage yield was investigated from 2 to 10 min based on the central composite design (CCD) run order. The results indicated that when extraction time increases, mucilage removal also increases as shown in Fig. 3 (D). This is because the solvent's penetration into the plant matrix is improved, and the mass transfer rate increases with prolonged contact between solvent and cladode while keeping other variables constant, which improves yield [41]. However, the increase in extraction time beyond 9 min while keeping other variables constant implies a decrease in yield, which may be caused by the needed component's deterioration in addition to the time and solvent used during the extraction process.

In order to obtain the highest yield of the desired polysaccharides without losing their fundamental composition, a research report states that microwave power and extraction time should be balanced during microwave-assisted extraction, meaning that at lower microwave power, high extraction time is needed and at higher power, low extraction time is needed [41]. The maximum yield was attained at these optimized values of around 9 min while keeping other parameters constant, and this result was slightly higher than the result of [41]. This may be caused by changes in the solvent, the solid-to-liquid ratio, the efficiency of the equipment, the chemical structure of the cladode, and environmental factors. Therefore, for this study 506 W microwave power and around 9 min extraction time gives highest yield.

#### 3.2.1 Interaction effect of microwave power and extraction time on yield

The interaction effect of microwave power and extraction time has a significant effect (p < 0.05) on the mucilage extraction yield. By increasing the surface area between the solvent and the cladode matrix and delivering an extended period of extraction time, the microwave power (A) and extraction time (D) increase the mucilage extraction yield. Because the matrix is exposed to radiation for extended periods of time, the microwave irradiation time increases, which accelerates the desorption of mucilage from the cladode cell wall [38]. The components that are required were not entirely removed from the plant matrix due to the shorter extraction times and lower microwave power.

**Fig. 4:**
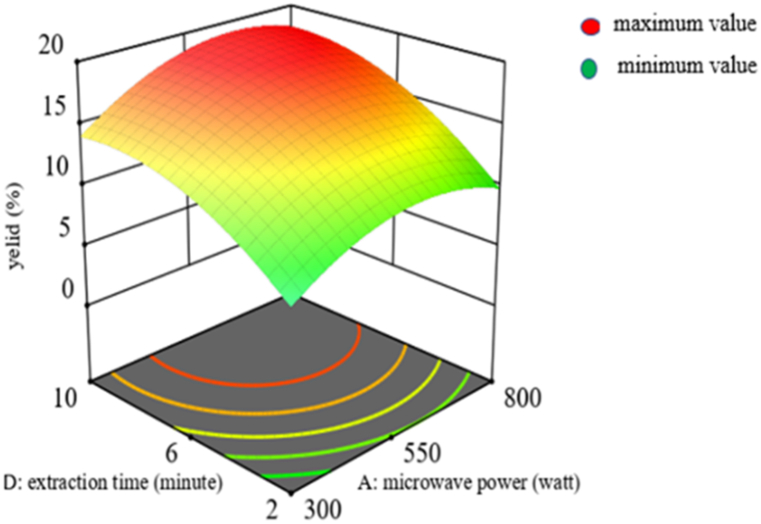
Interaction effect of microwave power and extraction time on mucilage yield.

On the other hand, lengthening the extraction process and increasing the microwave power may cause the fundamental structure of the mucilage to deteriorate, which could reduce the yield. This study confirmed that, the ideal extraction time and microwave power were 9 min and 506 W, respectively, as shown in Fig. 4. Therefore, as microwave power and extraction time increase, the yield of mucilage becomes enhanced until it reaches this optimum value. A similar trend was observed in the previous finding with similar extraction technique and different plant species [38].

#### 3.2.2 Interaction effect of concentration and solid-liquid ratio on mucilage yield

**Fig. 5:**
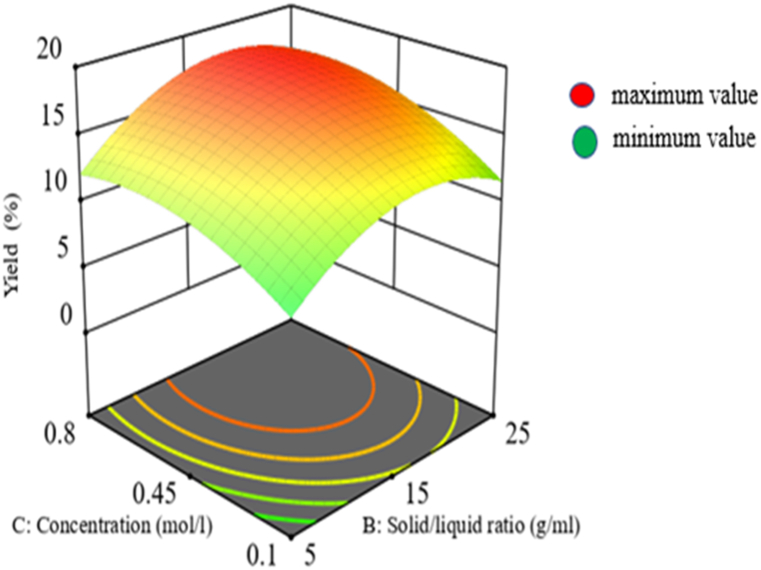
Interaction effect of concentration and solid liquid ratio on yield.

The interaction effect of concentration of sodium hydroxide and solid-liquid ratio have a significant (p < 0.05) effect on mucilage yield based on the analysis of variance response. According to the model equation, the yield is positively impacted by the interaction between the solid-liquid ratio (B) and sodium hydroxide concentration (C), as shown in Fig. 5. This suggests that the response, or mucilage yield, also improved when the sodium hydroxide concentration and the solid-to-liquid ratio increased. This might be a result of the desired component's improved solubility and disintegration of the cladode cell wall. The yield of mucilage is reduced at lower concentrations of sodium hydroxide and solid-to-liquid ratios because there is less solvent penetration into the plant matrix. At higher levels of both parameters, mucilage may degrade, and there may be a financial impact. Therefore, the interaction impact of these two independent factors should be optimized in order to obtain the optimum yield without composition degradation. So, with all other variables held constant, the greatest yield in this investigation was obtained at 0.606 mol/L of sodium hydroxide concentration and 1:20 of solid-liquid ratio.

#### 3.2.3 Interaction effect of extraction time and solid-to-liquid ratio on yield

**Fig. 6:**
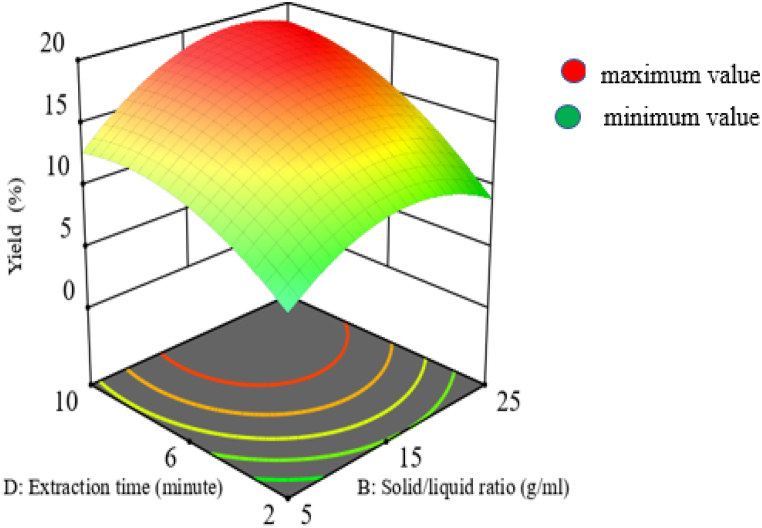
Interaction effect of extraction time and solid-liquid ratio on yield.

Based on the results of an analysis of variance using SPSS 25, the interaction effect of extraction time and solid-to-liquid ratio also has a significant impact (P < 0.05) on the yield of mucilage. According to the coded equation, the solid to liquid ratio and the interaction of the extraction duration have a favorable impact on the mucilage yield [38]. This shows that increasing the extraction time and solid-to-liquid ratio also increases the yield of mucilage since doing so improves the solubility of the required components from the plant matrix as shown in Fig. 6. Even so, increasing the solid to liquid ratio or the extraction duration above the recommended levels may cause the targeted component to degrade and have an adverse financial impact. The greatest yield in this investigation was achieved at a solid-to-liquid ratio of 1:20 and a time interval of 9 min, all other factors being held constant. Similar trends was observed in the finding with similar extraction technique [38].

### 3.3 Optimization on mucilage extraction yield

**Fig. 7:**
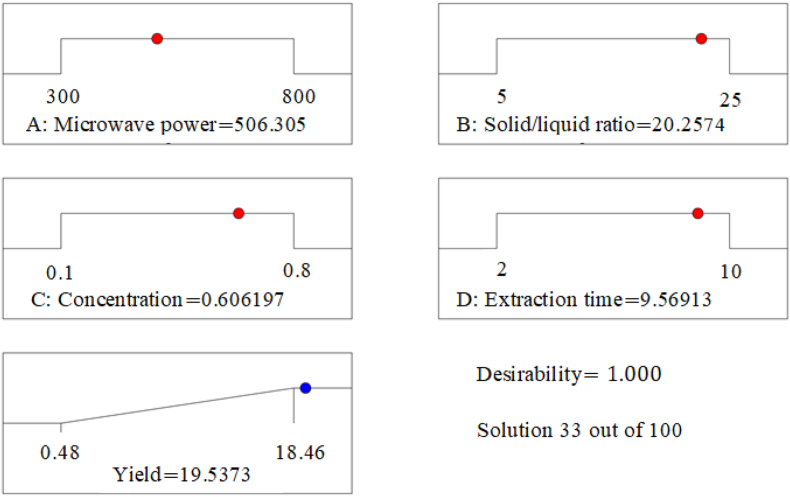
Diagram for selection of optimum values.

This study examined the extraction of mucilage from the Ethiopian cactus plant species (*Opuntia Ficus Indica*) using microwave-aided extraction procedures with sodium hydroxide solution as a solvent. Since this extraction technique is highly efficient, it reduces the extraction time needed and the amount of solvent used when compared to other conventional techniques [38,41]. At lower values of all independent variables’ mucilage cannot extracted effectively, where as they go to the maximum values may lead to degradation, so optimization is needed for better extraction. At the optimum level of independent variables, the maximum mucilage extraction yield of 17.15 % was obtained. By maximizing the response and assigning independent variables to a range, the software delivers 100 predicted solutions. By considering the cost, time, and composition of the necessary components, the microwave power of 506 W, the solid-liquid ratio of 1:20, the concentration of sodium hydroxide 0.606 %, the 9-min extraction time, and the yield of 19.54 with 1 desirability were chosen from this 100-predicted solution, as shown in Fig. 7 (A-D).

The validation was conducted at this optimized parameter to check the predicted value by conducting the experiment, and 17.15 % of mucilage was obtained. This is reasonable and in agreement with the predicted value at a 90 % confidence level. This result strongly deviated from the results reported in Refs. [26,42]. The difference could result from the surroundings, the kind of soil, the age of the cladode, the sampling period, or seasonal variations at the time the sample was taken and others may be responsible for the deviation [43]. However, this result was better than the one examined by Ref. [41], which found that 8.13 % and 6.93 % from cactus (*Opuntia Ficus Indica*) by using conventional extraction procedures and microwave aided extraction, respectively. This variation can be caused by the solvent used and the characteristics of cladode, which are influenced by environmental factors and different types of soil. The result of the experiment was in line with the result that obtained in Ref. [44] with similar cactus species. As a result, without knowing how cladode is used, a large amount of mucilage has been discharged into the environment in our locality. Since cactus fruit can be eaten, the oldest cladode is discarded into the environment unless it has been used by someone to feed cattle by burning the spine. Therefore, this study may be used as a reference for transforming these kinds of debris into valuable components and as a source of raw materials for many manufacturing sectors.

### 3.4 Characterization of mucilage powder

#### 3.4.1 Physicochemical properties of mucilage

The physicochemical properties of mucilage, such as solubility, moisture content, swelling power, crude protein content, ash content, fat content, water holding capacity, and carbohydrate content, were determined to know the properties of mucilage (Table 2). As shown Table 2, the physicochemical properties of powdered mucilage were presented in the range of previous literature values.

**Table 2:** Physicochemical properties of powdered mucilage from cladodes.

Parameters	Characteristic values		References
	Present study	Literatures	
**PH**	6.3 ± 0.99	5–7.5	[27,42,45,46]
**Solubility (%)**	20.4 ± 1.6	3–86	[24,27,47,48]
**Moisture content (%)**	13.58 ± 0.35	3–33	[24,[49], [50], [51]]
**Swelling power (%)**	32.3 ± 0.31	1–73	[27,47,48]
**Water holding cap.(g/g)**	8.58 ± 0.59	2–34	[24,47,50]
**Protein content (%)**	8.58 ± 0.13	1.2–14	[30,51,52]
**Ash content (%)**	6.67 ± 0.15	1.2–15	[30,50,51,53]
**Fat content (%)**	5.12 ± 0.88	1–2	[30,51]
**Carbohydrate content (%)**	66.2 ± 0.45	19–87.7	[30,51,52]

The pH of mucilage powder indicated that it is probably near to being too neutral, which suggests that mucilage has no negative impacts or harmful characteristics. However, the outcome was just on the edge of an acidic zone, which suggests the existence of galacturonic or uronic acid [38] and also confirmed by FTIR spectra of mucilage (Fig. 16) of absorption peaks of carbonyl (⁓1605 cm^−1^), hydroxyl (⁓3280 cm^−1^), and C–O stretching (⁓1023 cm^−1^) which may support the presence of galacturonic or uronic acid. The result is in agreement with similar plant species as well as organs [45]. Due to the presence of multiple hydroxyl groups throughout the entire molecule and confirmation from the analysis of FTIR spectra (Fig. 15), the results of moisture content, solubility revealed the hydrophilic features of mucilage [24]. These characteristics may make mucilage exceedingly brittle and thermally unstable when used for the development of films. The result of moisture content was strongly deviated from the previous report [54], this may be due to the environmental factors, seasonal variation as well as extraction methods. The swelling power and water holding capacity results were revealed that mucilage has a good water uptake ability [27]. In addition to this the nature of cladode, which resists draught by holding water in the inner regions of cladode via mucilage, was responsible for the water holding capacity and swelling power, the result was in agreement with the previous report [24] with different plant species and different extraction technique as well as types of solvent. Furthermore, the existence of such a high concentration of fat, protein, and carbohydrates suggested that mucilage can be used as a useful polysaccharide that could be employed in the production of other important components and the result was in agreement with the result [30] with another source of plant. The carbohydrate content and protein content of the present study were in agreement with the previous study [55]. As can be seen Table 2, the ash content value was strongly deviated from the result [27], this may be due to cladode characteristics, and composition of mucilage. In summary, the physicochemical characteristics results indicated that the major composition of mucilage is a carbohydrate.

### 3.5 Development of mucilage-based films

**Fig. 8:**
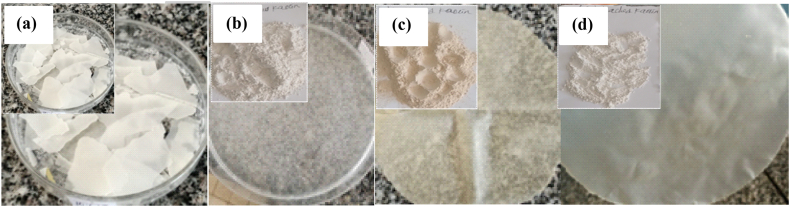
Development of mucilage-based films: (a) mucilage with distilled water, (b) mucilage, 5 % raw kaolin, 2 % urea, and 20 % glycerol, (c) mucilage, 5 % calcined kaolin, 2 % urea, 20 % glycerol and (d) mucilage, 5 % acid-leached kaolin, 2 % urea and 20 % glycerol.

Several lab-based preliminary works were carried out in the laboratory to make biodegradable films from cactus mucilage. As seen in Fig. 8(a), the result of mixing pure mucilage with distilled water was very fragile in similar working conditions. This may be due to the absence of plasticizers and crosslinking agents as well as the presence of strong intramolecular interaction with the mucilage molecule. While 5 % raw kaolin, 2 % urea, and 20 % glycerol were used as reinforcing materials, crosslinking agents, and plasticizing agents, respectively, using mucilage as the main ingredient, the resulting product cracked on the Petri dish plate rather than being of acceptable quality (Fig. 8(b)). This could be due to the presence of iron, which can cause a side reaction and change the way the film looks, or may be due to the presence of volatile or non-volatile impurities linked to the kaolin's surface. Moreover, when 5 % calcined kaolin was used with 2 % urea as a crosslinking agent and 20 % glycerol as a plasticizer with a similar procedure, the product was better relative to the raw kaolin, but still there was precipitate on the film surface, which led to a less attractive film surface in addition to weak mechanical properties (Fig. 8(c)). To enhance its properties, kaolin was leached with 2 M oxalic acid to remove iron from the kaolin, and the film was prepared by using 5 % acid-leached kaolin with 2 % urea and 20 % glycerol as crosslinking agents and plasticizers, respectively and hence film was very attractive based on physical observation (Fig. 8(d)). This is because the plasticizer weakens the strong intramolecular interactions between the molecules of mucilage, while urea crosslinks mucilage and kaolin via carbonyl groups and amine groups, which results in the formation of a strong hydrogen bond between mucilage and acid-leached kaolin via the two urea functional groups.

After deciding on the reinforcing material, the effect of glycerol and acid-leached kaolin on various film physicochemical properties was investigated based on the full factorial experimental design run order. Since the practical use of biodegradable plastic films depends significantly on their physicochemical properties. Hence, biodegradable plastic films with different compositions under similar conditions were developed to optimize the mechanical and biodegradability properties of the resultant films. The variance analysis indicated that the amount of glycerol and acid-leached kaolin significantly affects (p < 0.05) all the film physicochemical characteristics.

#### 3.5.1 Effect of glycerol and acid-leached kaolin on the moisture content of the films

**Fig. 9:**
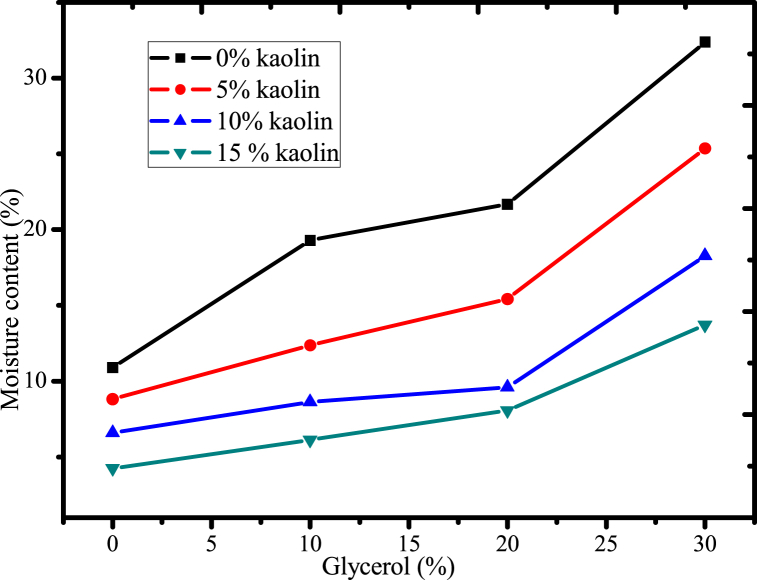
Effect of glycerol and acid-leached kaolin on the moisture content of films.

The moisture content of the films has a significant role in the practical application of biodegradable plastic films. Thus, the moisture content needs to be reduced to enhance the strength of the plastic [56]. As indicated in Fig. 9, the concentration of glycerol increased from 0 % to 30 % w/w relative to the mass of mucilage, increasing the moisture content of the plastic film. This is because glycerol's hygroscopic characteristics strengthen the film's sensitivity to water and enhance its hydrophilic properties of the bioplastic film due to the presence of hydroxyl groups, enabling the formation of hydrogen bonds with water molecules and the inclusion of water into the structure [14]. Moreover, excessive use of glycerol can lead to a decrease in mechanical strength and barrier properties of the film, as well as an increased risk of microbial growth. Therefore, it is important to carefully balance the amount of glycerol used in a film formulation to achieve the desired properties without compromising its performance. Similar results were also observed in the development of starch-based biodegradable films [57].

However, the addition of acid-leached kaolin reinforcing material cross-linked with urea, reduced moisture content from 32 % to 5 % when its concentration increased from 0 % to 15 %. When urea-cross-linked kaolin is added to the film, it forms a network of cross-links with the polymer chains via inter- and intramolecular interaction, which can reduce the moisture content of the film. The cross-linking process creates a barrier that prevents water molecules from entering the film, and limits the free hydroxyl group interaction with water, this might be reduced its moisture content of the film. This can be beneficial in applications where the film needs to maintain its shape and integrity in humid or wet environments [34]. Moreover, acid-leached kaolin crosslinked with urea also reduces the hygroscopicity of the film by interacting with the hydroxyl groups of mucilage, which are responsible for hydrophilicity, this might improve the hydrophobic properties of biodegradable films. Similar trends were observed in the starch based biodegradable film reinforced with silver-kaolin filler conducted by Ref. [56]. Therefore, urea cross-linked acid-leached kaolin can be a potential reinforcing material to reduce moisture content of the film for better practical application of biodegradable films.

#### 3.5.2 Effect of glycerol and acid -leached kaolin on films thickness

**Fig. 10:**
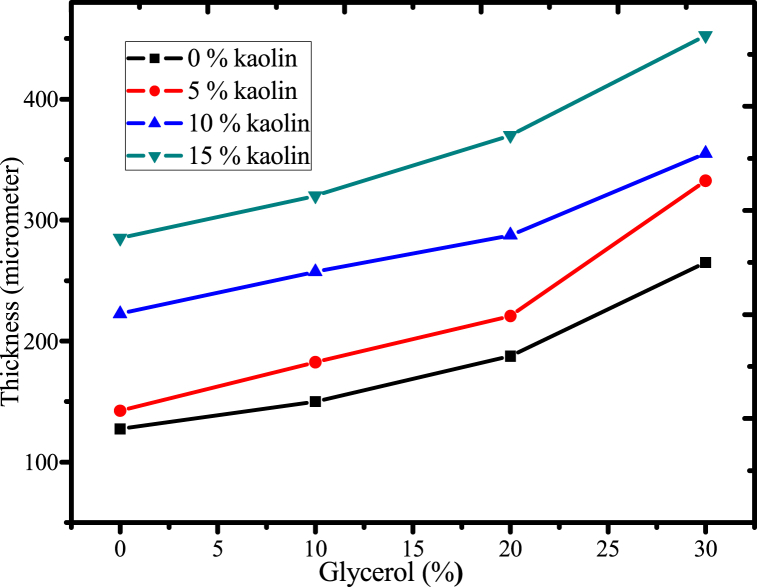
Effect of glycerol and acid-leached kaolin on film thickness.

The amount of glycerol and reinforcing material impacted the formed plastic film's thickness, which was measured using a digital micrometer in four duplicates. The results (Fig. 10) showed that increasing the concentration of glycerol and acid-leached kaolin greatly increased the thickness of biodegradable plastic film. This is because the amount of glycerol in the polymer matrix has increased, and the incorporation of the reinforcing material increases the amount of solid content in the polymer matrix, causing the film to be thicker than when there is a lower concentration of filler [56]. Furthermore, as the concentration of plasticizer rise from 0 % to 30 %, the thickness of the film also increased. This may be caused by the role that plasticizers play in the disruption and restructuring of intermolecular polymer chain networks, which resulted in the creation of more free volumes and, ultimately, thicker film thickness. Also, during the crosslinking process, the urea molecules react with the functional groups of the kaolin and mucilage, creating new chemical bonds that link the components together. This can result in a tighter packing of the particles, which can lead to an increase in the overall thickness of the biodegradable film. A similar trend was observed in the findings of the development of starch-based films by using glycerol and micro-pottery clay [14]. In summary, the amount of plasticizer and reinforcing material, acid-leached kaolin, enhanced the thickness of the biodegradable plastic film.

#### 3.5.3 Effect of glycerol and acid-leached kaolin on the solubility of the films

**Fig. 11:**
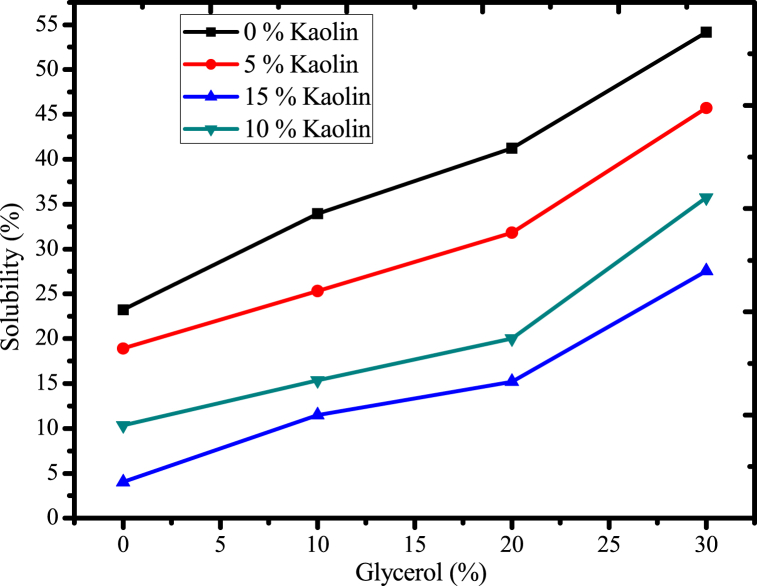
Effect of glycerol and acid-leached kaolin on films solubility.

Solubility plays a crucial role in the practical use of biodegradable plastic films, particularly in packaging applications, since it increases the brittleness of the plastic film and the spoilage of the desired product or food that is packed inside. Therefore, lowering the solubility of bioplastic increases the perceived value of those plastics. This study assessed the impact of glycerol and reinforcing material on film solubility, and the results are displayed as indicated in Fig. 11. The results of this study proved that increasing the concentration of glycerol plasticizer improved the solubility of films as well as their flexibility and uniformity in the film matrix [58]. The hydrophilic properties of mucilage [35] and glycerol makes it easier for plastic film to dissolve in water [14]. By reducing the strong intramolecular interaction of polymer chains, raising the concentration of glycerol improves the film's solubility and attraction to water [14]. Hence, glycerol can be easily introduced between polymer chains since it has the lowest molecular weight and the most hydrophilic nature when compared to the other polyols this leads to the dissolution of polymer in water [14,18]. It can also improve the flexibility and elasticity of the film. This can be beneficial in applications where the film needs to conform to irregular shapes or withstand repeated bending and folding.

However, the solubility of the film in water has decreased when acid-leached kaolin has been incorporated into the polymer matrix in amounts ranging from 0 % to 15 %, as shown in Fig. 11. This is due to the possibility that urea-crosslinked, acid-leached kaolin could reduce free hydroxyl groups and limit the interaction of the polymer with water since urea is more reactive than glycerol and water. According to the previous report [59], the carbonyl and amine groups of urea interacted favorably with the siloxane and alumina surfaces of kaolin, and this interaction continued with the hydroxyl groups of cactus mucilage to reduce the solubility of the bioplastic film. This pattern was consistent with the information [56]. Therefore, incorporating reinforcing material has played a significant role in reducing such types of defects in the film development process [58].

#### 3.5.4 Effect of glycerol and acid-leached kaolin on transparency of the films

**Fig. 12:**
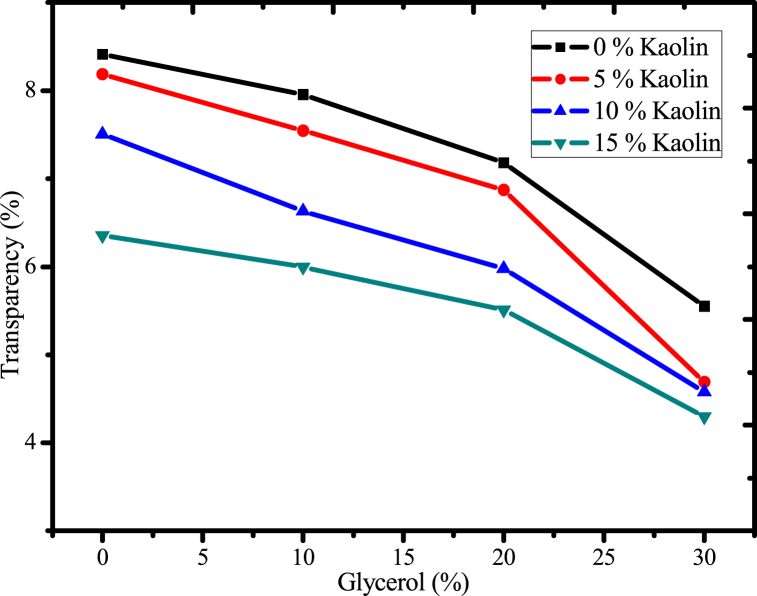
Effect of glycerol and acid-leached kaolin on transparency of the films.

In this study, the effect of glycerol as a plasticizer and acid-leached kaolin as a reinforcing material on the film that was produced was examined by adjusting the concentration relative to mucilage mass, and the outcome was illustrated as shown in Fig. 12. Transparency is essential in various packaging applications to increase consumer acceptance of the product [35]. It exhibits the homogeneity of the film-forming solution and the clarity of the plastic film [60]. However, depending on the function of plastics, such as when it's necessary to pack light-sensitive items, increasing opaqueness or decreasing transparency also play a crucial role [36]. Packaging with high or low brightness, depending on consumer preferences,the marketing industry has extensively used opacity as a technique to symbolize both a sort of information and an emotional connection between the consumer and the product [35]. The outcome showed that the film's transparency decreased as the glycerol concentration increased; this could be because the film's thickness increased. This means when the film thickness increases due to increment of amount of glycerol the bioplastic film may absorb or scatter more light rather than transparent as a result the opaqueness may enhance [60]. Furthermore, this tendency was anticipated as a result of the rise in dry matter content and the glycerol molecules' occupation of the intermolecular spaces or gaps in the polymer matrices [35]. A similar result was observed from starch-based biodegradable films with similar plasticizers [35]. When the concentration of acid-leached kaolin was increased from 0 % to 15 %, the transparency of the resulting film was reduced from 8.8 % to 4.7 %; this may be because the enhancement of the amount of solid in the film also lead to the raising of thickness, as a result of which light may be absorbed or scattered by the entire material of the film matrix. In addition to this the crosslinking process creates a more compact structure, which makes it more difficult for light to pass through the material. In general, the amount of glycerol and acid-leached kaolin were reduced the transparency of the biodegradable films.

#### 3.5.5 Effect of glycerol and acid-leached kaolin on tensile strength of the films

**Fig. 13:**
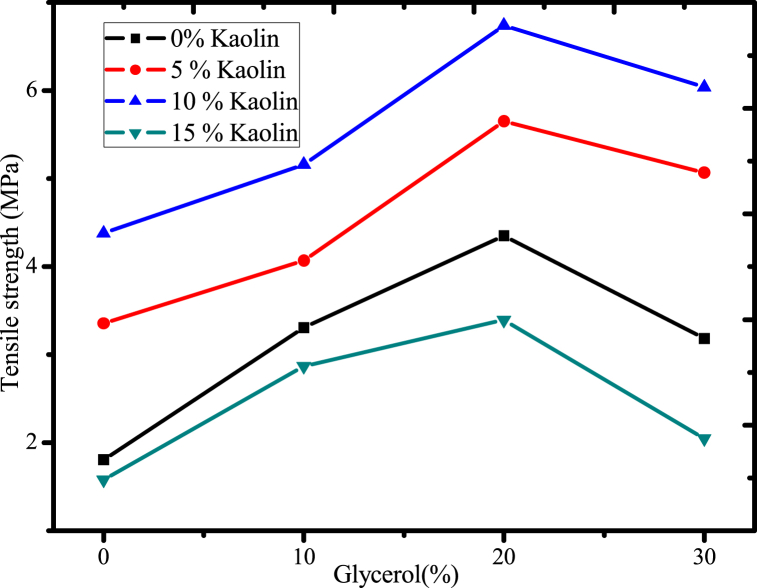
Effect of glycerol and acid-leached kaolin on tensile strength of the films.

The chemical composition of the polymer matrix and other additives play a significant role in the strength of biodegradable plastics [61,62]. This study examined the relationship between glycerol concentration, acid-leached kaolin, and tensile strength to produce the best bioplastic film with outstanding characteristics, as shown in Fig. 13. This finding showed that the tensile strength decreased as the glycerol concentration increased from 10 % to 30 %. This is because the addition of glycerol changes the way that polymers interact with one another by lowering the main ingredient's glass transition temperature; as a result, the polymer's intermolecular interaction is diminished, which significantly reduces its tensile strength [58]. However, the minimum or negligible amount of glycerol results in the rigidity of the polymer matrix [58]. This showed that the ideal proportion of glycerol was essential to the development of biodegradable plastics with good mechanical properties [58]. Also, the outcome this work showed that the plastic film's tensile strength increased when the concentration of acid-leached kaolin increased from 0 % to 10 % (Fig. 13). This may be because of urea's interaction with acid-leached kaolin improves the film's strength by restricting the interaction of mucilage's hydroxyl groups with water. Additionally, the chemical crosslinking agent urea was used, and it may interact simultaneously with the hydroxyl groups of mucilage and acid-leached kaolin via carbonyl or amine groups and lead to the formation of a strong hydrogen bond, which enhances the tensile strength of the film. Moreover, when the urea crosslinked kaolin is added to the biodegradable film, it forms a network of particles that can distribute stress throughout the film, making it more resistant to tearing and breaking. The crosslinking of the urea with the kaolin also helps to improve the adhesion between the kaolin particles and the biodegradable polymer matrix, further enhancing the mechanical properties of the films.

However, when the amount of acid-leached kaolin was increased from 10 % to 15 %, the tensile strength decreased. This was caused by the formation of agglomeration on the polymer matrix or by the weak intramolecular interaction of mucilage and acid-leached kaolin, which may have occurred and resulted in the formation of voids between the polymer's entire molecule [14]. The maximum tensile strength of 6.74 MPa was attained at 10 % acid-leached kaolin and 20 % glycerol (Fig. 13). The result was higher than the previous report [56], which was on gelatin-based film reinforced with silver kaolin composite, on semolina flour-based films reinforced with nano-kaolin [58]. In addition, the result was approximately in agreement with starch-based film reinforced with micro-pottery clay [14]. However, the result was strongly deviated from the result reported in mucilage-based biodegradable film reinforced with polyvinyl alcohol [58], may be due to the difference in the load cell (5000 N). In addition, this biodegradable plastic film's tensile strength was higher than that of the mucilage-based bioplastic film reinforced with agar (5.3 MPa) using a similar film-forming process [35]. In general, the tensile strength of reinforced film was improved by 77 % compared to the control bioplastic film when using readily available, cost effective and promising reinforcement material of acid-leached kaolin.

#### 3.5.6 Effect of glycerol and acid-leached kaolin on elongation of the film

**Fig. 14:**
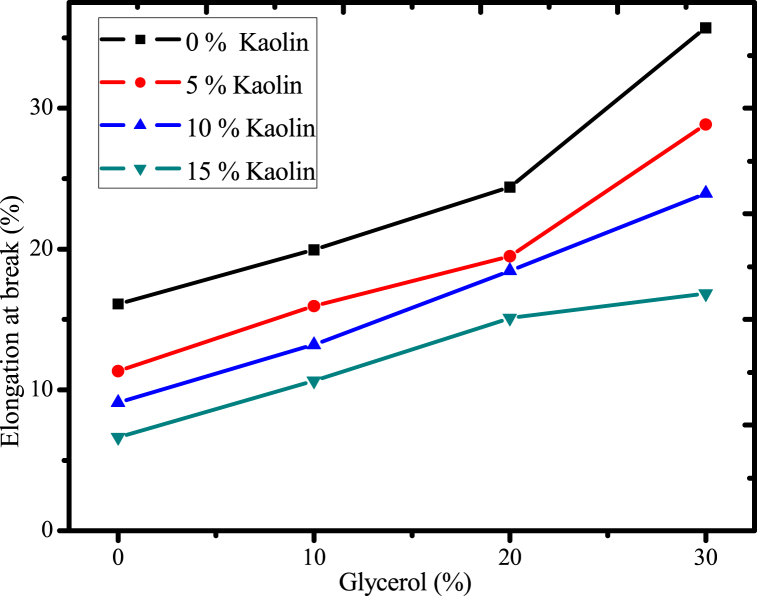
Effect of glycerol and acid-leached kaolin on elongation at break film.

The effect of glycerol concentration and acid-leached kaolin crosslinked with urea on the elongation at break was investigated based on a full factorial experimental run order (Fig. 14). Basically, in most cases, tensile strength (TS) has a reverse relationship to elongation at break (EAB), which measures the flexibility of films. As a result, it was clear that increasing the content of glycerol as a plasticizer also increased the elongation at break or flexibility of the biodegradable film. This is because glycerol decreases the intermolecular interactions of the polymer matrix, and because it has a lower molecular weight, it is easier to penetrate the matrix of the polymer. This improves the mobility of the molecules, which increases the flexibility of the final film [35,58]. On the other side, the bio plastic film's elongation at break was altered by the addition of acid-leached kaolin cross-linked with urea as a reinforcing ingredient. The elongation at break of the film decreases as the acid-leached kaolin concentration rises from 0 % to 15 %. This is because urea-cross-linked kaolin can decrease the elongation at break of biodegradable film because the crosslinking process creates a rigid network of particles within the film. This network restricts the movement of the polymer chains, making it harder for the film to stretch and deform before breaking. In addition, there is also the possibility of the formation of strong hydrogen bonds, electrostatic interactions, and Vander wall interactions, which make films more rigid and reduce their elasticity. A similar observation was noted in the finding of a starch-based biodegradable film using nano kaolin as a filler [58]. In general, the reinforced film's elongation at break was improved by 53 % over the control film due to the addition of glycerol as a plasticizer.

**Fig. 15:**
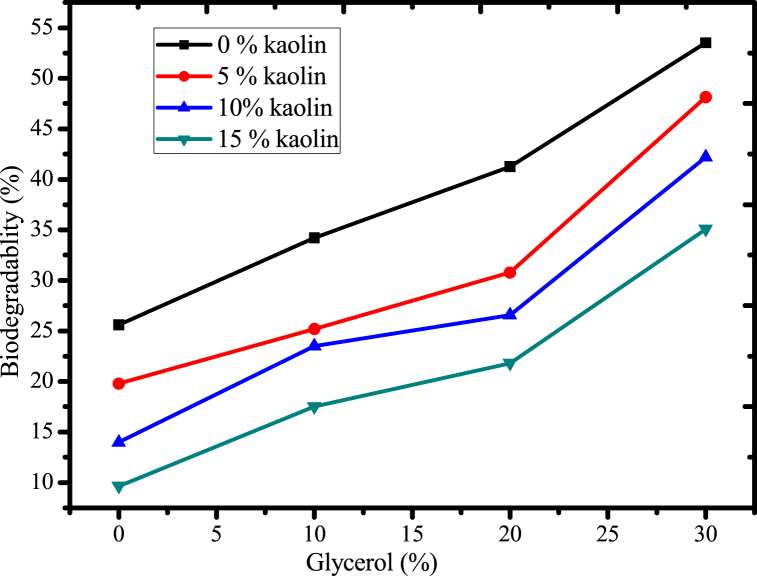
Effect of glycerol and acid leached kaolin on biodegradability of the films.

#### 3.5.7 Effect of glycerol and acid-leached kaolin on biodegradability of the film

The effect of glycerol concentration and acid leached kaolin on biodegradability were conducted based on soil burial method and the results are presented in Fig. 15. The results showed that, when glycerol concentration increased, biodegradability also increased due to the hydrophilic nature of glycerol which increases moisture content. The development of microorganisms is facilitated by an increase in moisture content or water affinity, which enhances biodegradation. Similar trend was observed in the finding of starch-based bio plastics [63]. An increase in glycerol concentration might reduce the strong interaction of the polymer's matrix, weakened the intermolecular interactions, and therefore, microorganisms may degrade substances easily. Hydrophilic fillers may enhance degradability, which increases the film's water affinity and promotes the biodegradation process [14].

In this study, when the concentration of acid leached kaolin is increased from 0 % to 15 %, the biodegradability is reduced due to the formation of strong intermolecular or intramolecular interactions and decrease in moisture content of the polymer matrix caused through the hydrophobic nature of acid-leached kaolin. Additionally, the complexity of the polymer matrix may increase as a result of incorporation of acid leached kaolin, which reduces biodegradability since it requires several different microorganisms to attack a variety of chemical linkages. Also, urea cross-linked kaolin reduces the biodegradability of the film because it creates a more stable and resistant structure that is less susceptible to degradation by microorganisms. The crosslinking process makes the film more compact and less porous, which limits the access of microorganisms to the polymer chains and reduces their ability to break down the materials. Moreover, the presence of kaolin particles can further hinder biodegradation by acting as a physical barrier that prevents microorganisms from reaching the polymer chains. The maximum biodegradability 53 % was attained at 30 % glycerol concentration, but the optimization was performed based on tensile strength value and at maximum value of this parameter the degradability was 26.57 % within 30 days degradation time in soil burial method.

### 3.6 Surface functional group analysis of films using FTIR

**Fig. 16:**
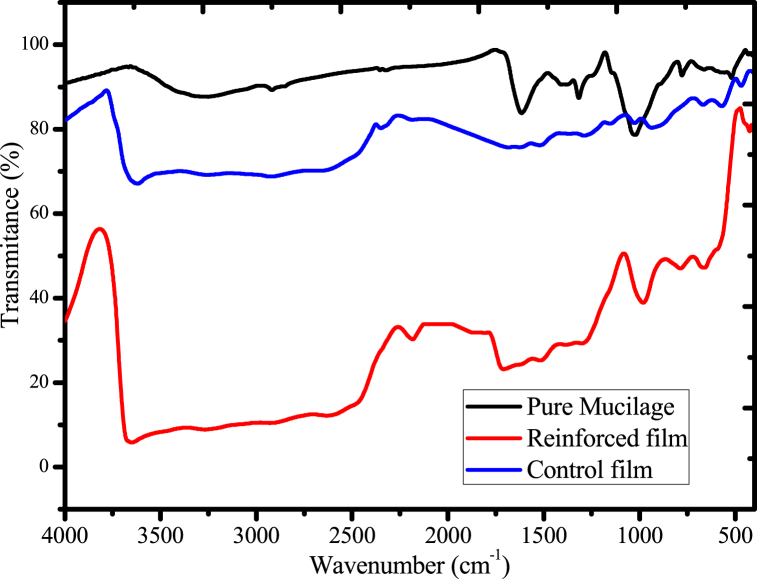
FTIR spectra of pure mucilage, control and reinforced films.

The surface characteristics of mucilage and mucilage-based films are presented in Fig. 16. For mucilage (Fig. 16), broad absorption peak at ⁓3280 cm^−1^ were observed, indicates the presence of a hydroxyl groups (mucilage contains many hydroxyl groups) in their large carbohydrate molecules [27]. Moreover, absorption peaks of asymmetric/symmetric C–H stretching at (⁓2923 cm^−1^), C–O stretching (⁓1023 cm^−1^), C=O stretching due to the presence of a small amount of uronic acid or N–H bending due to the presence of proteins (⁓1605 cm^−1^) were observed [34].

The surface functional groups mucilage-based biodegradable films (control and reinforced) were also presented in Fig. 16. Broad absorption peaks at ⁓3612 cm^−1^ (control) and ⁓3664 cm^−1^ (reinforced) were associated due to the presence of O–H/N–H stretching [35]. Also, the peak at ⁓2190 cm^−1^ was observed in both films may be due to the formation of N≡C and/or C≡C stretching during the removal of water upon films development process [64]. Also, peaks at ⁓1550 cm^−1^ (control) and 1529 cm^−1^ (reinforced) were observed due the presence of C=O and C–N/C=N stretching [59]. Moreover, characteristics peaks at ⁓912 cm^−1^ (control) and ⁓990 cm^−1^ (reinforced) were associated may be due the presence of N–H bending, O–H deformation and Si–O/Si–*O*–Al stretching vibrations (reinforced, incorporation of acid leached kaolin) of films [[65], [66], [67]]. The slight absorption peaks position shift or diminish might be due to the incorporation of urea as crosslinking agent and acid leached kaolin as reinforcing material and thus, formation of strong hydrogen bonds between the hydroxyl group of mucilage and the alumina or siloxane surface of acid-leached kaolin crosslinked with the two functional groups of the urea molecule. Additionally, the percent transmittance of the reinforced film was lower than that of the control film, indicating that the reinforcing material had been incorporated into the polymer matrix.

### 3.7 Determination of thermal properties of films

**Fig. 17:**
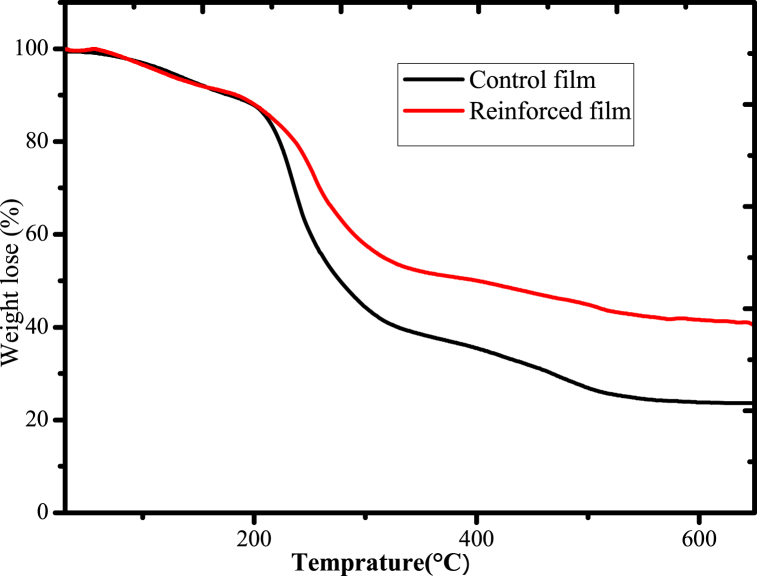
TGA thermogram for mucilage-based biodegradable films.

Thermogravimetric analysis was carried out in the temperature range of ambient temperature to 900 °C with a heating rate of 20 °C in a nitrogen environment to evaluate the thermal characteristics of mucilage and mucilage based biodegradable films (Fig. 17). For mucilage, the results revealed that there were three fundamental deterioration thermograms, with the first ranging from 60 to 140 °C with a 5 % mass loss, the second from 180 °C to 400 °C with 60 % mass loss and 240–400 °C with 60 % mass loss and the third from 680 to 810 °C with 75 % mass loss (Fig. 17) agreeing with the previous reports [35,68,69]**.** The first, second and third stages denotes the elimination of moisture and bound water from the mucilage's surface, beginning of mucilage disintegration of internal structures and caused by the presence of minerals since the mucilage contains minerals, especially calcium, respectively [34,69]. The thermogravimetric investigation of the mucilage powder obtained from the Ethiopian cactus species indicated that it can be thermally stable up to 180 °C and may be a viable raw material for a variety of industries, particularly the packaging industry.

The thermal characteristics mucilage-based biodegradable films were carried out from room temperature to 700 °C (Fig. 17). Two degradation peaks at temperatures between 60 °C to 180 °C and 206 °C–400 °C were presented for both films (Fig. 17). The initial degradation showed that the polymer matrix for both biodegradable films had been cleaned of the adsorbed water on its internal and external surfaces and also possibly the removal of plasticizers (glycerol) [32]. The second step was described as the thermal behavior of both bioplastic films and the degradation of polysaccharides. However, some improvements in the thermal stability of reinforcing film were noted, and the temperatures at which deterioration occurred with corresponding mass loss for both bioplastic films were reported (Table 3).

**Table 3:** Mass loss for control and reinforced films at the same temperatures.

Temperature(°C)	Mass loss for reinforced film (%)	Mass loss for control film (%)
**222**	15.2	19.5
**242**	20.1	33.1
**260**	30.3	44.89
**500**	54.56	72.8
**624**	58.85	76.4

As shown in Table 3, the percentage mass loss of reinforced film was lower when compared to the mass loss of control film at the same temperature due to the effect of acid-leached kaolin as a reinforcing material enhances the thermal stability of the biodegradable films. This may be caused by the intermolecular strong hydrogen bonds that are formed between mucilage and acid-leached kaolin crosslinked with urea molecule. Considering the results of an investigation into the intercalation of urea with kaolin [59], the interaction between urea and kaolin was energetically favorable leading to in the formation of strong hydrogen bonds via amine groups that can act as hydrogen donors and carbonyl that can act as hydrogen acceptors with siloxane and alumina surface of kaolinite clay. Both the mechanical and thermal characteristics are considerably enhanced by this strong bond formation. Therefore, in order to alleviate the bottleneck issues with biodegradable film, acid-leached kaolin cross-linked with urea can be used as potential reinforcement material for the development of bioplastics.

### 3.8 Morphological analysis of mucilage-based biodegradable films

**Fig. 18:**
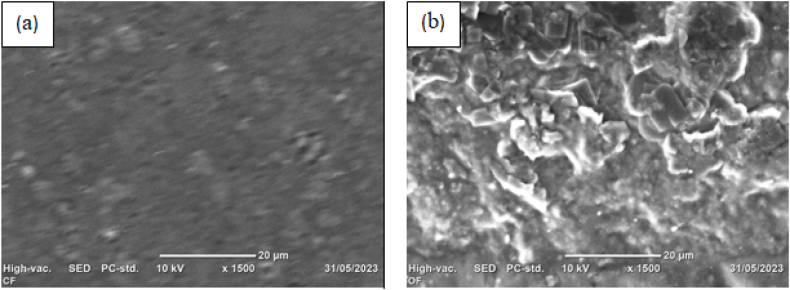
SEM image: (a) control and (b) reinforced films.

Scanning electron microscope (SEM) was used to examine the surface smoothness and roughness of control and reinforced films Fig. 18 (a) and (b) respectively. As Fig. 18 (b), the reinforced film was found to be rougher than the control film, presumably due to the incorporation of acid-leached kaolin in the polymer matrix. In addition, the reinforced film image displays white particles, which represent the distribution of kaolin that has been acid-leached and crosslinked with urea molecules. The increasing film thickness might also be to blame for the film's increased roughness, and a similar tendency was observed in the literature [58]. The scanning electron microscope (SEM) result of pure cactus mucilage was investigated and it was irregular, indicating the defects of pure mucilage film [35]. However, in this investigation, the control film had a smoother surface than the reinforced film, as opposed to the latter's rough surface Fig. 18 (a). The reason for this is that, the control film departed from the pure mucilage SEM picture because it contained 2 % urea as a crosslinking agent. This shows that urea and mucilage interacted very well, yet the control film's tensile strength was lower than that of the reinforced film; this results from the lack of reinforcing material.

### 3.9 Analysis of crystallinity of films

**Fig. 19:**
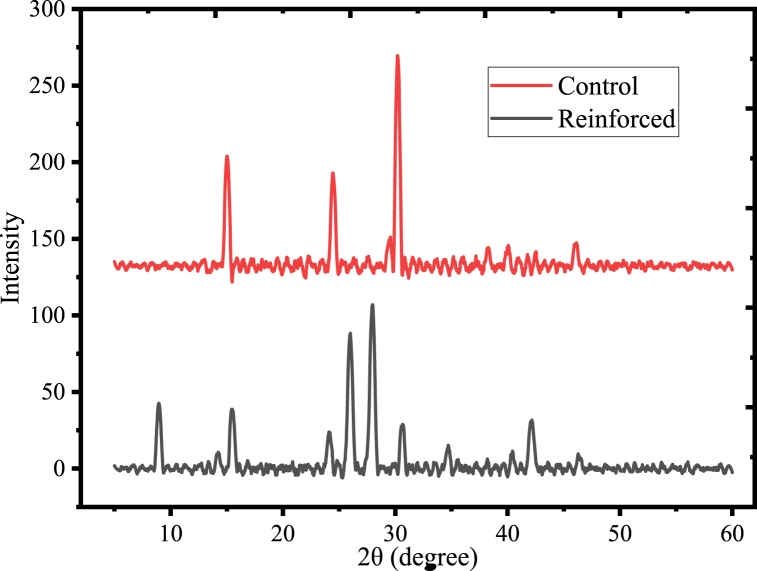
XRD result for mucilage-based biodegradable films.

The crystallinity of the control and reinforced films were conducted using an X-ray diffractometer and the results are given in Fig. 19. The results revealed that, the new crystalline peak was noted at 2 θ values of 9.7^o^, 25-30^o^, 35^o^, and 43^o^ in the diffractogram of reinforced film, indicates the incorporation of acid leached kaolin from the polymer matrix. The crystalline peak at 2 θ values of 14.3^o^ and 30^o^ for both control and reinforced films might be the presence of mucilage [70]. Furthermore, the peak that is noted at 9.8^o^ in the reinforcing film may indicate the intercalation of urea with acid-leached kaolin, and the peak that appeared at 25.6^o^ in the reinforcing material may also indicate the presence of kaolin in the polymer matrix. The result indicated that both control and reinforced film show semi-crystalline properties. However, the crystallinities of control film and reinforced film were 8.9 % and 15.35 %, respectively. This indicates that the reinforced film was more crystalline than the control film. This may be due to the formation of a strong hydrogen bond or Vander walls force during the film development process via acid-leached kaolin and mucilage crosslinked with urea molecules. A similar trend was reported in the previous study [58]. Moreover, the peak at 2 θ values of 24.6^o^ in the control film was reduced in intensity in the reinforced film, indicates how the incorporation of acid-leached kaolin reduces the crystallinity of the control film (Fig. 19).

## 4 Conclusion and future outlook

In this study, cactus (*Opuntia Ficus Indica*) cladodes were used as a source of polymeric mucilage extracted via microwave-assisted extraction technique. The mucilage yield and physicochemical properties were analyzed under optimal experimental parameters: microwave power, extraction time, solid-liquid (mucilage-sodium hydroxide solution) ratio, and sodium hydroxide concentration. This mucilage was converted into biodegradable plastics using glycerol as a plasticizer and acid-leached kaolin cross-linked with urea as a reinforcing material. A full factorial experimental design run order was used to determine how the glycerol and acid-leached kaolin affected various physicochemical properties of the biodegradable film. The results indicated that tensile strength increases as acid-leached kaolin increases due to the formation of strong hydrogen bonding and attained a maximum value of 6.74 MPa from the combination of 20 % glycerol and 10 % acid-leached kaolin. The biodegradable film's elasticity, solubility, and moisture content all rise with the addition of glycerol. The intermolecular interaction in the polymer matrix is reduced as a result of glycerol's hydrophilic properties and low molecular weight. Moreover, the thermal properties of biodegradable plastics were improved by incorporating acid-leached kaolin cross-linked with urea, as supported by the results of FTIR and SEM images. In general, cactus mucilage as the main ingredient and acid-leached kaolin cross-linked with urea as the reinforcing material can be an ingredient for the development of biodegradable plastics. The biodegradable polymers developed for this study may be used for packaging application. In the future, additional characterization such as air or oxygen permeability, water vapor permeability and rheological properties should be needed.

## Funding

This work has been funded by Bahir Dar Institute of Technology, 10.13039/501100005872Bahir Dar University, Ethiopia (www.bdu.edu.it).

## Data availability

Data will be made available on request.

## CRediT authorship contribution statement

**Alebel Abebaw Teshager:** Writing – review & editing, Writing – original draft, Software, Methodology, Investigation, Formal analysis, Data curation, Conceptualization. **Minaleshewa Atlabachew:** Writing – review & editing, Supervision. **Adugna Nigatu Alene:** Writing – review & editing, Software, Methodology.

## Declaration of competing interest

All authors have read and understood the policy of the competing interests and declare that there are no competing interests.
